# Revealing the nature of active sites in electrocatalysis

**DOI:** 10.1039/c9sc02654a

**Published:** 2019-07-23

**Authors:** Batyr Garlyyev, Johannes Fichtner, Oriol Piqué, Oliver Schneider, Aliaksandr S. Bandarenka, Federico Calle-Vallejo

**Affiliations:** a Physics of Energy Conversion and Storage , Technical University of Munich , James-Franck-Straße 1 , 85748 Garching , Germany . Email: bandarenka@ph.tum.de; b Departament de Ciència de Materials i Química Fisica , Institut de Química Teòrica i Computacional (IQTCUB) , Universitat de Barcelona , Martí i Franquès 1 , 08028 Barcelona , Spain . Email: f.calle.vallejo@ub.edu; c Electrochemical Research Group , Technische Universität München , Schleißheimerstraße 90a , 85748 Garching , Germany; d Catalysis Research Center , TUM , Ernst-Otto-Fischer-Straße 1 , 85748 Garching , Germany

## Abstract

Heterogeneous electrocatalysis plays a central role in the development of sustainable, carbon-neutral pathways for energy provision and the production of various chemicals. It determines the overall efficiency of electrochemical devices that involve catalysis at the electrode/electrolyte interface. In this perspective, we discuss key aspects for the identification of active centers at the surface of electrocatalysts and important factors that influence them. The role of the surface structure, nanoparticle shape/size and the electrolyte composition in the resulting catalytic performance is of particular interest in this work. We highlight challenges that from our point of view need to be tackled, and provide guidelines for the design of “real life” electrocatalysts for renewable energy provision systems as well as for the production of industrially important compounds.

## Abstract 2

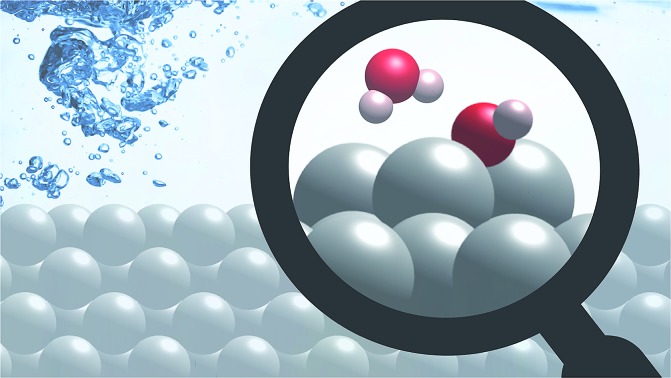
In this perspective, key aspects for the identification, design and optimization of active centers at the surface of electrocatalysts are analyzed.

## Introduction

Many would agree that our lives today would be radically different without the Haber–Bosch process, as this revolutionary work in heterogeneous catalysis on nitrogen fixation enabled the fast growth of the world's population.^1^ Since then, landmark contributions were made especially by Sabatier,^2^ Langmuir,^3^ Taylor,^4^ Balandin^5^ and Ertl^6^ to understand chemical reactions at surfaces. To recognize the importance of catalytic reactions at solid surfaces, Nobel prizes were awarded to P. Sabatier for his work on hydrogenation reactions catalyzed by metals in 1912, to F. Haber for his research on ammonia synthesis in 1918, to I. Langmuir for the study about surface reactions and adsorption in 1932, and G. Ertl for his work on understanding of chemical processes at solid surfaces in 2007. Nowadays, heterogeneous catalysis partakes in the production of nearly 90% of the chemicals used in our daily life; and with the growing concerns about climate change, the development of sustainable, carbon-neutral pathways for the production of energy and industrially essential chemicals is paramount.^7^–^9^ Rationally increasing the efficiency of catalysts mainly depends on elucidation of the nature of active sites that control the overall catalytic performance.^10^–^12^

The idea that only several active sites at the catalyst surface control the overall catalyst performance was introduced by Taylor in 1925.^4^ Ever since, especially over the past decades, advances were made on the development of model single-crystal surfaces and ultra-high vacuum methodologies. These, in turn, enabled experimental studies of the electronic and structural properties of the catalytic centers on well-defined surfaces. In parallel, great advances in theoretical chemistry in combination with the rapid development of computer technologies led to the establishment of new approaches to understand and predict the reaction rates at different surface sites as a function of their composition and structure. In particular, density functional theory (DFT) has provided deep insight into heterogeneous catalysis as a whole.^7^*In situ* experimental and computational studies concur that catalytic activities are dictated by the nature of the active sites, which strongly depends on the materials' composition^13^–^22^ and surface structure,^23^–^26^ and the electrolyte composition.^27^–^31^

In this perspective, we analyze several fundamental issues related to the elucidation of the electrocatalysts' active centers and factors that modify their catalytic efficiency. Firstly, aspects related to the electronic structure of the active sites are briefly discussed; then the role of surface defects is considered, as it is pertinent to ask if surface defects are the only sites able to catalyze electrochemical reactions. Subsequently, we elaborate on how the activity changes with the size and shape of nanoparticles and whether the intrinsic activity can be controlled solely by adjusting the coordination environment of the active sites. Afterwards, the influence of the electrolyte composition, especially the role of the so-called spectator species, is analyzed. Finally, we mention the possibilities for the prediction of the selectivity and stability of catalytic active sites.

## Electronic structure of active sites

A suitable strategy to enhance the catalytic activity of pure transition-metal catalysts is to alloy them with other metals.^32^–^34^ In the following, we refer to ligand effects as those introduced by a guest (alloying) atom on a host substrate. More specifically, ligand effects refer to the modification of the adsorption energies and catalytic activities induced by the presence of an alloying atom in the proximities of the active sites. The alloying atoms are not in direct contact with the adsorbates, so that ligand effects are due to the modification of the active sites' electronic structure. On the other hand, geometric effects (discussed in the next section) appear when the spatial configuration of the active sites is changed, which can happen if (I) the interatomic distances of the active sites are stretched/contracted, or (II) atoms are added to the active sites or removed from them. In this context, a pure metal may only experience geometric effects, while an alloy can experience both electronic and geometric effects.

Different kinds of alloys have been explored in the literature, including bulk alloys (BAs), thin films (TFs), surface alloys (SAs) and near-surface alloys (NSAs). The variation in adsorption energies in BAs with respect to the respective pure metals is attributed to strain and ligand effects, while in TFs we only have strain effects—when thicker than 3 atomic layers—and in NSAs only ligand effects.

In the particular case of oxygen electroreduction (ORR, O_2_ + 4H^+^ + 4e^–^ → H_2_O), which requires large overpotentials and is of interest in proton-exchange membrane fuel cells (PEMFCs), it is well known that the ideal cathode catalyst should bind *OH intermediates more weakly than Pt(111) by approximately 0.1 to 0.15 eV: Δ*G*XOH – Δ*G*Pt(111)OH = (0.1–0.15) eV.^35^ Note in passing that this criterion usually applies to ORR electrocatalysts with Pt active sites, for which it is reasonable to assume that the prefactors are comparable.^36^ Following this energetic design principle, TFs of Pt on top of a Cu bulk were devised that display a 4–6 fold enhancement in ORR activity over pure Pt.^37^ Those catalysts seize the strain effects on the Pt layers, as Pt and Cu have appreciably different lattice constants. For Pt, compressive lattice strain weakens the binding of the surface to adsorbed intermediates, whereas tensile strain has the opposite effect.

Strasser and coworkers suggested that the high ORR activity of dealloyed PtCu_*x*_ could be attributed to the weakening of the adsorption energy of *OH *via* strain.^37^ The same effect causes the high activity of Pt-rare-earth alloys: after exposure to electrolyte, rapid dissolution of the non-noble component takes place due to the very high reactivity of rare earth metals, leaving behind a 5–6 layer thick, compressively strained layer of pure Pt.^20^,^38^–^40^ This layer shows a large ORR activity (a factor of 6 in surface-specific activity compared to pure Pt), and stabilizes the alloy bulk against further dissolution. Moreover, PtCo and PtNi alloys have shown promising results in terms of activity and long term stability.^41^–^43^

On the other hand, in NSAs solute atoms are only located in the proximities of the surface in submonolayer amounts and mostly induce ligand effects on the catalyst layer. Ligand effects are more pronounced when the solute atoms are at the subsurface (*i.e.* the second atomic layer), and are already negligible when they are located in the fourth atomic layer. Chorkendorff and co-workers have shown that subsurface Cu in Pt suitably weakens the binding of ORR intermediates, thereby inducing an 8-fold activity improvement.^32^ In addition, subsurface Cu atoms in Pt have been successfully tested for the hydrogen evolution reaction^33^ and CO oxidation,^34^ in view of the suitable modification of adsorption energies of the respective intermediates with respect to Pt(111).

**Fig. 1:**
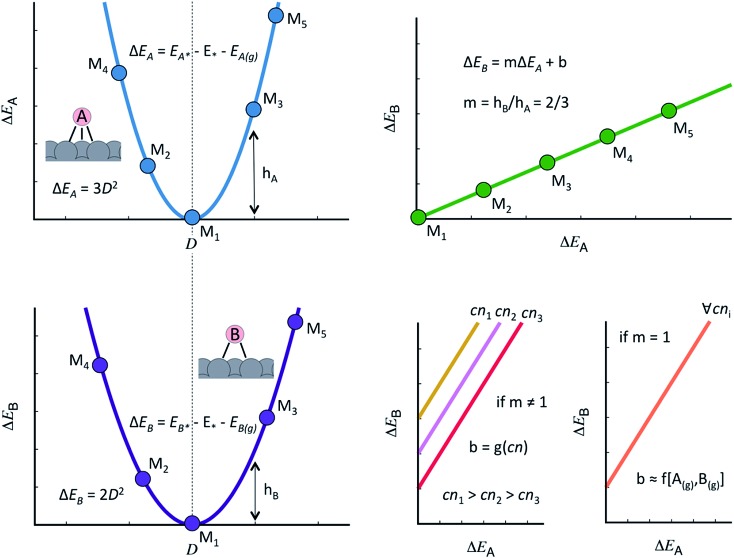
Generalities of adsorption-energy scaling relations. Left: the adsorption energies of species A (Δ*E*_A_, top) and B (Δ*E*_B_, bottom) for materials M_1_ to M_5_ scale with a hypothetical descriptor *D* and their minima coincide. A makes three bonds with the surface and B makes two. Upper right: because Δ*E*_A_ and Δ*E*_B_ scale similarly with descriptor *D* and the minima coincide, there is a linear scaling relation between them and the slope of the line is the ratio of the bonds made to the surface. Lower right: if the slope is not 1, the offset of the scaling relation depends on the coordination number of the active sites. Conversely, if the slope is 1, the offset is surface-independent and proportional to the gas-phase energetics of A and B.

The discovery of linear relations between sets of adsorbates similarly bound to transition metal active sites is one of the milestones of computational surface science and heterogeneous catalysis of the past fifteen years.^44^ Formally, when the adsorption energies of species B scale linearly with those of species A as: Δ*E*_B_ = *m*Δ*E*_A_ + *b*, *m* can be estimated as the ratio between the lack of bonds of species A and B to fulfill the octet rule, and *b* is a constant that depends on the coordination of the surface atoms,^45^,^46^ see Fig. 1. Such relations have also been shown to hold on surfaces of oxides, nitrides, sulfides, carbides, porphyrins and single-atom catalysts on graphite layers,^47^,^48^ among others. Moreover, scaling relations are not restricted to species occupying the same adsorption sites, and thus scalability may exist between, *e.g.*, adsorbates on top sites and adsorbates on bridge or hollow sites. The general condition for scalability is that the extrema of the curves that correlate adsorption energies with the electronic structure be the same for species A and B.^45^,^46^ This is illustrated in Fig. 1 where, for simplicity, we assume a quadratic dependence with a hypothetical electronic-structure descriptor *D*. Before continuing, we emphasize that the dependence needs not be quadratic, it can have any other functionality. In practice, *D* may be a simple descriptor such as the number of valence electrons of the metal atoms at the adsorption sites,^45^,^47^,^48^ work functions, electronic charges on the adsorbates, d-band centers, or a more sophisticated descriptor such as the crystal orbital overlap population (coop) or the crystal orbital Hamilton population.^49^

As shown in Fig. 1, the offset *b* depends on the value of the slope:^46^,^50^ if *m* ≠ 1, the offset is proportional to the coordination of the adsorption sites. This is the case of *OH *vs.* *O, the slope of which is 1/2, as *O lacks two electrons to fulfill the octet rule and the oxygen atom in *OH lacks one. If *m* = 1, the offset is surface independent and depends on the gas-phase energetics of the species involved. This is the case of *OOH *vs.* *OH, the slope of which is 1.

Note in passing that there are also scaling relations between adsorption energies and other types of energetics, for example bulk energies of formation.^51^ Besides, scaling relations with negative slopes also exist between certain adsorbates. Conventional scaling relations are formed between electronegative adsorbates such as F, O, N, and C. Conversely, anomalous scaling relations with negative slopes are established between those and less electronegative adsorbates such as B.^49^

The downside of scaling relations is that they allegedly limit the efficiency of electrocatalysts. For instance, the scaling relation between *OOH *vs.* *OH is thought to limit the oxygen evolution and reduction reactions (OER, ORR): while the energetic separation of those intermediates should ideally be 2.46 eV, on most catalysts it is ∼3.20 eV.^52^,^53^ Another example is the scaling relation between *CO and *CHO, which is supposedly responsible for the high overpotentials of CO_2_ reduction to CH_4_.^54^ However, a word of warning is needed here, as recent works have shown that: (a) breaking the *OOH *vs.* *OH scaling relation is a necessary yet insufficient condition to achieve ideal OER/ORR electrocatalysts,^55^,^138^ and such breaking can actually increase the overpotential. (b) *CHO is not always formed during CO_2_ reduction.^50^,^56^ (c) For some materials, *CHO formation does not influence the overpotential.^57^ In brief, breaking a given scaling relation is not an infallible recipe for the optimization of all electrocatalysts. Instead, one should focus on the specific reaction steps and intermediates that are problematic.

Thermodynamically, the only general guideline for optimal performance is electrocatalytic symmetry,^58^,^138^ which is achieved when all the reaction steps have free energies numerically equal to the equilibrium potential (or its additive inverse). For example, the OER (H_2_O → O_2_ + 4H^+^ + 4e^–^) has an equilibrium potential *E*^0^ = 1.23 V, and the free energies of the four proton–electron transfers are each 1.23 eV on the ideal catalyst. In this context, a metric for symmetry is provided by the electrochemical-step symmetry index (ESSI), the minimization of which effectively corresponds to lower calculated overpotentials.^55^,^138^ It is defined as: 
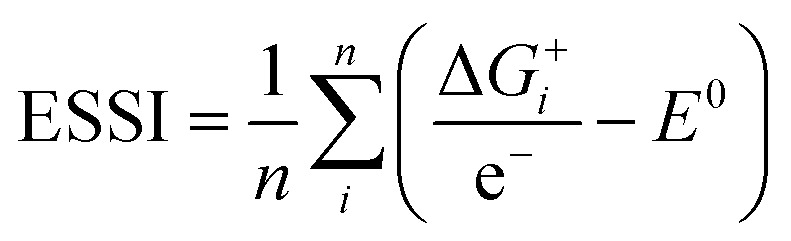
, where 
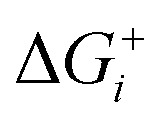
 are the reaction free energies for which Δ*G*_*i*_ ≥ e^–^*E*^0^ (as only those can control the potential), and *n* is the number of electrochemical steps for which Δ*G*_*i*_ ≥ e^–^*E*^0^.

## Geometric structure of the active sites

**Fig. 2:**
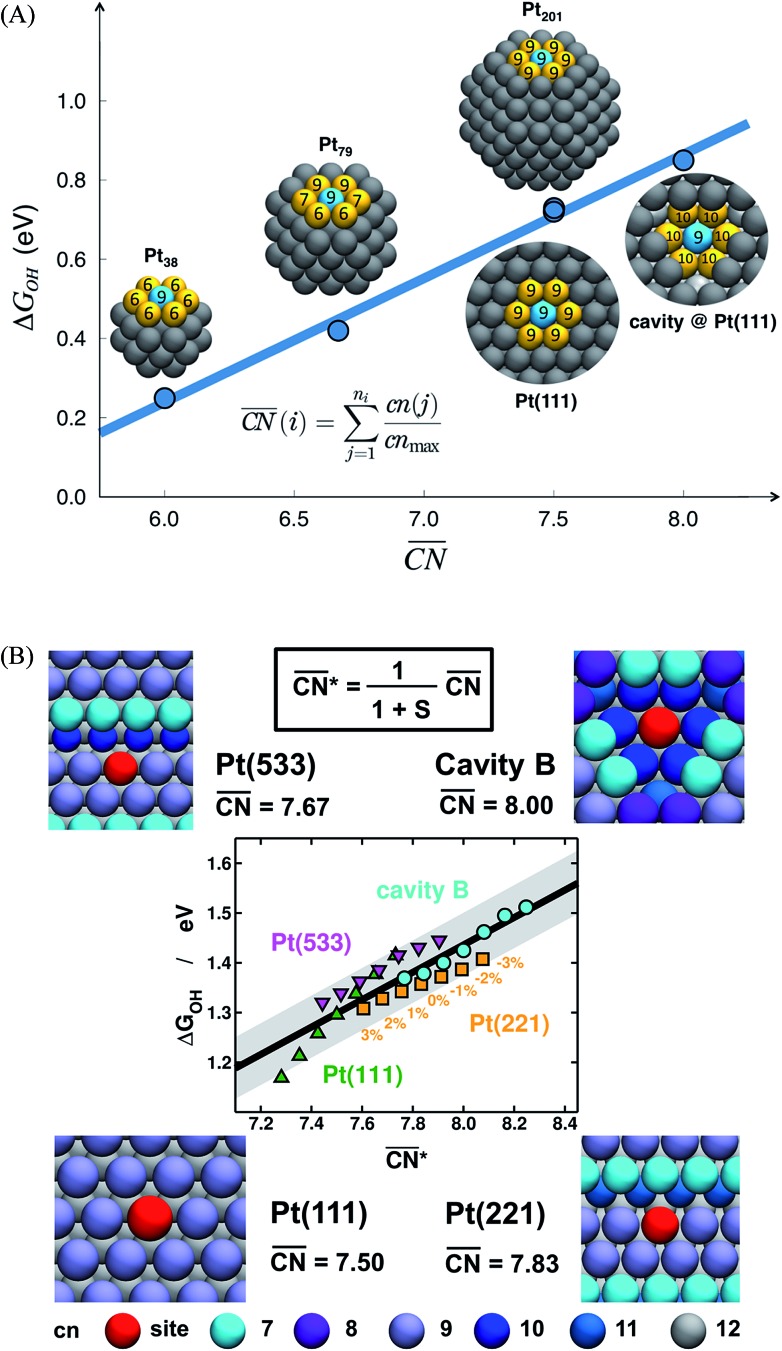
Adsorption energies as a function of generalized coordination numbers 
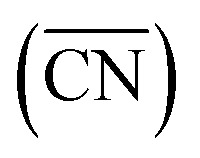
 for various surface sites. (A) Trends in *OH adsorption energies for Pt sites (in blue) with 9 nearest neighbors (yellow) but different coordination of such neighbors. Values taken from ref. 67 and 73. (B) Trends in *OH adsorption energies for Pt sites with 9 nearest neighbors but differently coordinated second nearest neighbors and with positive (stretching) and negative (contracting) strain. Adapted from ref. 62 with permission from John Wiley & Sons.

The spatial arrangement of the active sites influences their adsorption behavior and catalytic activity. The most common and probably simplest geometric descriptor is the conventional coordination number (cn), which for metals is nothing but the number of atoms located in the proximities of atom *i*, taking as a reference the interatomic distance observed in the bulk. Usually, there is a (nearly) linear relationship between cn and adsorption energies, in which high coordination corresponds to weak adsorption energies, and low coordination corresponds to strong adsorption energies.^46^,^50^,^59^ The trends are clearer for extended surfaces, as finite-size effects in nanoparticles may induce large deviations, in particular for small particles.^60^,^61^ Finite-size effects are illustrated in Fig. 2A, where all of the blue sites on nanoparticles and extended surfaces have cn = 9 but their adsorption energies of *OH differ by more than 0.5 eV. The differences arise not from the number of nearest neighbors but from their respective coordination numbers, which vary appreciably.

Fig. 2A also shows that generalized coordination numbers 
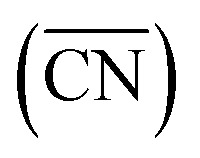
 can capture the trends in adsorption energies for extended surfaces and nanoparticles,^61^ which is achieved by taking into account not only the number of nearest neighbors but also their respective conventional coordination number:
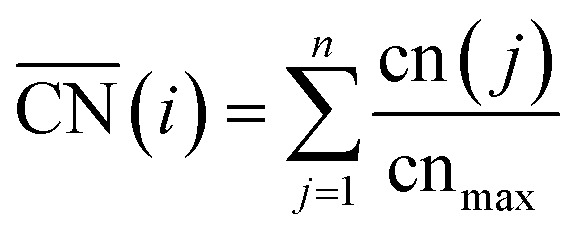


Moreover, the following analytical relationship between 
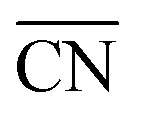
 and d-band centers has been shown to exist, which has important implications^61^ in surface science and catalysis:
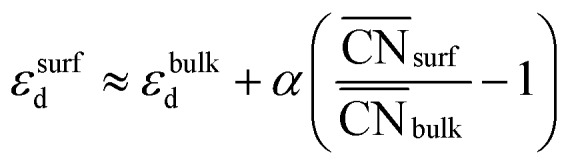


This equation connects the electronic and geometric structures of a (late) transition metal by means of the d-band center of the surface sites (*ε*surfd) of the material, the d-band center in the bulk (*ε*bulkd), the generalized coordination number of the surface sites 
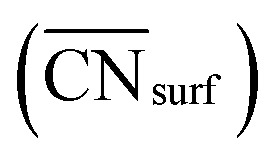
, and that of the bulk (
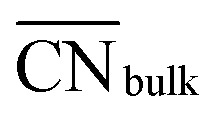
, which is 12 for an fcc crystal). In this formula, *α* is a material-specific constant that depends on the cohesive energy and the average occupancy of the d-band (*α* ≈ 1.44 eV for Pt). The link between the electronic and geometric structures of materials suggests that geometric descriptors may as well be used to design enhanced (electro)catalysts, as we will show later in this review.

Before, we mentioned that strain is one of the most widely used strategies for the enhancement of Pt-based alloys for the ORR.^20^,^37^–^40^ In the context of geometric descriptors, strain (*S*) can be regarded as a manifestation of generalized coordination in which the number of nearest neighbors is constant but the interatomic distances are different. This consideration leads to a strain-sensitive version of generalized coordination numbers 
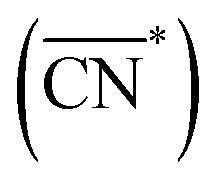
:^62^
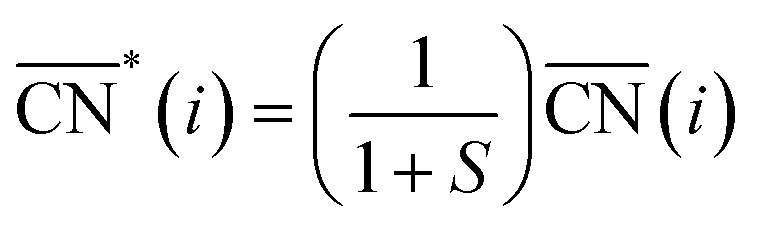


The trends in adsorption energies of *OH for stretched (*S* > 0) and contracted (*S* < 0) Pt sites are shown in Fig. 2B as a function of 
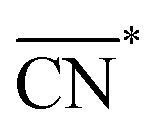
. In agreement with experiments, a contraction of the Pt–Pt bonds leads to a weakening of the *OH adsorption energies on Pt, and the opposite is true when stretching. Note that the *S* factor is the average degree of strain (in %) of the interatomic distances with respect to the bulk of the material. Recent works on Pt alloys with lanthanide elements have shown that *S* can be as large as 6%.^20^ However, at the surface layer the actual strain is lower, so that *S* is not larger than 3%.^63^

Before concluding this section, it is worth noting that generalized coordination numbers can be calculated on metals other than Pt^64^–^67^ and have been used to analyze trends for a variety of (electro)catalytic reactions.^33^,^64^,^66^–^70^

## The role of surface defects

Nowadays, the rational design of heterogeneous electrocatalysts is mainly based on the modification of surface properties. In the case of the ORR on Pt, for example, numerous theoretical as well as experimental approaches have been used to modify the surface properties through *e.g.* alloying, size and shape effects. For instance, nearly spherical, unstrained Pt particles show theoretical maxima in mass activity for diameters around 1, 2 and 3 nm.^71^,^72^ These maxima are constrained by a narrow size distribution (±0.2 nm), which is difficult to transfer to real-life applications using conventional methods, although recent experimental results are rather encouraging.^72^ Therefore, it is desirable to provide further possibilities for tailoring surface properties.

A facile way to rationally improve activity by means of surface modifications is based on the generation of structural defects, leading to the introduction of particularly active surface sites. We stress here that not all sorts of defects improve the activity. To discern specifically active surface configurations one can use 
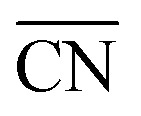
 61 and its associated coordination-activity plots.^62^,^67^–^70^

As said before, for the ORR on Pt the activity is typically correlated to the adsorption energy of *OH, which according to the Sabatier principle should not be bound too strong nor too weak in order to enable an efficient electrocatalysis. Instead of the usual correlation between activity and binding properties, within the 
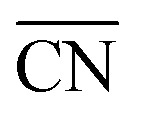
 approach the correlations are based on the connection between coordination environment and adsorption energies as described in the previous section. This enables a fast prediction of the ideal geometrical environment of the active sites compared to the time-consuming calculations of electronic-structure descriptors.^71^

**Fig. 3:**
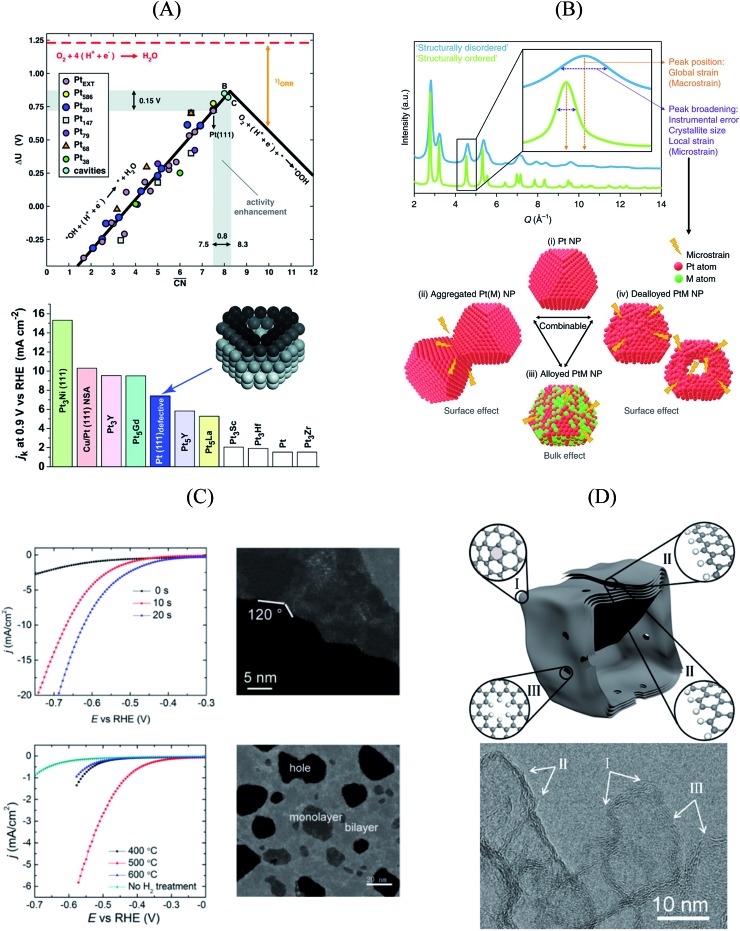
(A) Coordination-activity plot of various Pt surfaces (top), indicating a close-to-optimum coordination environment of surface cavities. A comparison of the defective Pt(111) surface with plain Pt shows a ∼3.5 times increased activity (bottom). Figures reproduced from ref. 67 with permission from American Association for the Advancement of Science. (B) WAXS spectrum peak broadening, induced by microstrain. The presence of microstrain in (i) conventional Pt nanoparticles, (ii) aggregated particles, (iii) alloyed Pt–M nanoparticles and (iv) dealloyed Pt–M nanoparticles is indicated in the Figure. Figures reproduced from ref. 77 with permission from Nature Publishing Group. (C) HER activity of oxygen plasma treated (top left) and H_2_ annealed (bottom left) MoS_2_, indicating an increased performance using both methods. The activity increase is caused by the generation of step-like edge sites in the basal plane of the catalyst, as shown in the corresponding TEM image (top and bottom right). Figures reproduced from ref. 78 with permission from American Chemical Society. (D) Schematic description (top) and TEM image (bottom) of possible defective sites on carbon nanocages, improving the ORR activity of the catalyst. Figures reproduced from ref. 81 with permission from American Chemical Society.

As shown in Fig. 3A, Pt(111) has a 
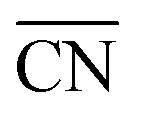
 of 7.5, while the optimum is located at ∼8.3. A way to bring the value closer to the optimum is to modify the coordination of the nearest neighbors by increasing the number of second nearest neighbors (see Fig. 2). This can be done, for example, by creating cavities through the removal of surface layer atoms. Experimental investigations at such defect sites validated the theoretical predictions, leading to 
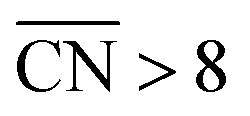
. For example, the dealloying of a Cu/Pt(111) surface alloy showed that the increase in activity of the Pt skin can be traced back to the presence of surface cavities. Moreover, it turns out that, in terms of activity, certain Pt structures are able to outperform Pt(111) by a factor of ∼3.5 and even some of the best Pt-based ORR catalysts known (Fig. 3A).

Studies on nanoparticles revealed that for typical convex particle shapes a maximum 
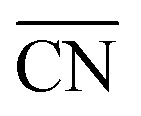
 of 7.5 can be reached, due to the sole presence of (111) and (100) surface facets.^73^ Conversely, concave surface defect sites, as *e.g.* present at the boundary of coalescent nanoparticles, can be used to increase the generalized coordination number, thereby suitably weakening the *OH binding. Experimental evidence of the enhanced activity of concave sites towards the ORR has been shown by Xia's group^74^ and Strasser's group,^75^ among others. However, shape-specific synthesis of such structures is still a challenging task. Similar active sites were also simulated at hollow, frame-like nanoparticles, matching with experimental observations provided by *e.g.* Dubau *et al.*^76^

An experimental way to assess the impact of defective sites on the ORR performance of bimetallic electrocatalysts (*e.g.* PtNi-based nanoalloys) was recently proposed by Chattot *et al.*^77^ In their work, surface distortion is introduced as a novel descriptor correlating electrochemical activities and properties such as structural surface defects and strain. A combination of different X-ray and microscopic techniques was exploited to prove that, in fact, initial structural disorder of bimetallic electrocatalysts leads to improved efficiency (that is, combination of initial activity and long-term stability) in simulated hydrogen fuel cell operation. This metric can be derived from the so-called “microstrain” introduced into the catalyst as a result of atomic disorder, which was determined by Rietveld refinement of synchrotron wide-angle X-ray scattering (WAXS) patterns (Fig. 3B).

Essentially, they found that while catalysts based on an ordered, surface-science-based approach (“downscaling” of active bulk surfaces) contain a maximum number of certain catalytic active sites, rapid degradation under operating conditions could be observed. However, particles with structural surface defects tend to have various surface sites, including a few particularly active ones, enabling both high initial activity and stability. These results pave the way for the rational design of particularly active and stable catalysts, turning away from well-established design criteria based on the investigation of extended surfaces.

The influence of disordered surfaces can be further extended to other important reactions taking place in energy conversion and storage devices. Investigating MoS_2_, a promising future catalyst for the hydrogen evolution reaction (HER), Ye *et al.* improved the activity of a MoS_2_ monolayer using a facile technique.^78^ Both oxygen plasma exposure and annealing in hydrogen atmosphere were separately utilized to introduce defects to the basal plane of the catalyst, which is rather inactive towards the HER compared to the defect-rich catalyst edges. Herein, released edge-like structures greatly promote the HER activity of MoS_2_ in both cases, as indicated in Fig. 3C.

In the emerging field of non-precious metal electrocatalysis, surface defects also play an important role by replacing common dopants (such as *e.g.* N and S) as active sites. This is of particular interest, as carbon-based ORR materials doped with N and Fe or Co could drastically reduce the catalyst costs compared to currently used Pt nanoparticles.^79^,^80^ However, an activity and stability similar to Pt-based materials has to be achieved. For instance, Jiang *et al.* investigated the impact of different defect types on the activity of pure-carbon nanocages using both experimental and computational techniques.^81^ They observed that, in fact, pure carbon outperforms certain doped carbon materials in alkaline media, presupposing the presence of defective surface sites. In particular, pentagon structures on the hexagonal surface (Fig. 3D(I)) and “zigzag” edge sites (Fig. 3D(II)) have been found to increase the activity of the nanocages.

In general, surface defects can help increase the electrocatalytic activity of various materials. However, defects on the catalyst support can contribute to its degradation. For instance, high surface area carbon, used in hydrogen fuel-cell catalysts, contains a large number of structural defects, owed to its heterogeneous structure. During typical fuel-cell operation potentials and conditions, defective carbon sites tend to hydrolyze, forming unstable carbon oxides that promote fast degradation of the support material.^82^

In summary, defect engineering is a simple way to improve the electrocatalytic activity of a material. Computational models combined with experiments can be used to determine whether defects or terraces control the activity. If defects predominate, theory can ascertain whether the active sites are located at concave (high 
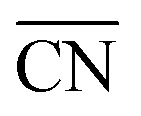
) or convex (low 
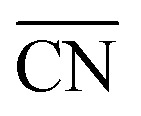
) defects. On Pt, for the ORR^62^,^67^,^73^ and the HER^68^ concave defects and strain increase the activity, while convex defects enhance CO oxidation.^69^ A particular case are Pt(100) terraces, which catalyze a variety of electrocatalytic reactions such as dimethyl ether oxidation and ammonia oxidation,^59^,^83^ and their activity is downgraded by defects.

It ought to be considered as well that defects could strongly affect durability, which is particularly true for carbon-based catalysts and support materials. Moreover, nanoparticle synthesis with suitable surface defects and good stability is still a challenging task. Thus, future research should find facile ways to specifically implement defects that grant control over the catalytic properties of nanostructures. In addition, simple catalyst design criteria should be available in order to reach maximal efficiency. In this order of ideas, geometric descriptors (such as generalized coordination numbers) are more recommendable than energetic descriptors (such as adsorption energies or band centers), as their connection to experiments is more straightforward. The ORR on Pt(111) illustrates this point: increasing the coordination of the active sites and weakening the *OH adsorption energy both lead to optimality. From an experimental standpoint, the former is probably more informative than the latter, as it contains information about the spatial configuration of the active sites. Energetic descriptors are, nonetheless, more widely used nowadays in computational electrocatalysis.^44^

## Size effects

The catalytic activity of metal nanoparticles has been investigated for nearly 4 decades. With the emergence of synthetic routes for size- and shape-controlled nanostructured particles in the late 20^th^ and early 21^st^ centuries by the groups of El-Sayed^84^,^85^ and Somorjai,^86^ measuring size- and shape-dependent activity studies was made possible. In electrocatalysis, the Strasser^11^ and Xia^74^ groups have driven the research focused on the electrocatalytic applications of size- and shape-controlled nanoparticles.

The catalytic activity of nanostructured particles is governed by a set of physical parameters including size, shape and geometric structure. In this subsection we will focus on the size-dependent activity^87^–^90^ of several nanostructured catalysts and the efforts made to locate the active sites within those.

**Fig. 4:**
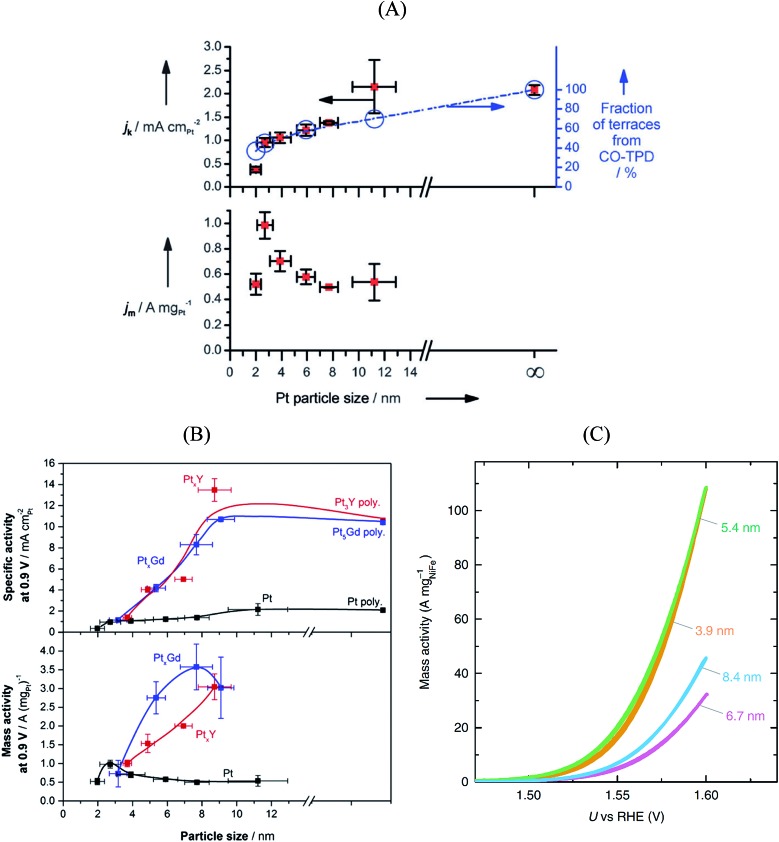
Mass and specific activities of (A) pure Pt nanoparticles and (B) Pt_*x*_Gd and Pt_*x*_Y nanoparticles as a function of the particle size. The activities at 0.9 V *vs.* RHE are extracted from cyclic voltammograms recorded in O_2_-saturated 0.1 M perchloric acid, at 1600 rpm, and the scan speed was 50 mV s^–1^. (A) and (B) reproduced from ref. 91 and 40 with permission from John Wiley & Sons and Elsevier, respectively. (C) Oxygen evolution reaction performance of NiFeO_*x*_H_*y*_ nanoparticles with various sizes. The cyclic voltammograms were recorded in N_2_-saturated 1 M KOH at 1600 rpm, and the scan speed was 10 mV s^–1^. Reproduced from ref. 94 with permission from Nature Publishing Group.

Platinum and Pt-group metals are known to be the best catalysts for the ORR in acid. The surface-area specific (*j*_k_ in mA cm^–2^) and mass-specific activity (*j*_m_ in A mg_Pt_^–1^) of Pt nanoparticles of different sizes (between 2–11 nm) towards the ORR is compared in Fig. 4A.^91^ The area-specific activity increases alongside the nanoparticle size and approaches bulk Pt surface activity near 11 nm. To locate the ORR active sites, temperature programmed CO desorption (CO-TPD) experiments were conducted in vacuum. From CO-TPD measurements the authors estimated the percentage of terraces present in a given nanoparticle. Previously, theoretical studies^92^ had predicted that the active sites were located at the terraces of the Pt surface and, as shown in Fig. 4A, the surface-specific activity of nanoparticles increases with the percentage of terraces. Briefly, the percentage of terrace atoms increases with the nanoparticle size and consequently the number of active sites. However, the activity per mass has an optimal surface-to-volume ratio and the nanoparticles with 3 nm size have shown the highest mass activity (see Fig. 4A). For alloyed Pt_*x*_Y^93^ and Pt_*x*_Gd^40^ nanoparticles the size dependence curve is similar to that of pure Pt nanoparticles; however, the highest activity is observed for the 8–9 nm nanoparticles. This is mainly because only beyond those dimensions a stable strained Pt skin can form, so that smaller particles are unstable and dealloy rather quickly.

In summary, the ORR active centers are located at the terraces of regular Pt nanoparticles and the interplay between surface area to mass determines the quantity of the active sites with nearly optimal binding energy towards the key reaction intermediate *OH, which determines to a large extent the overall performance of the electrocatalyst.

The OER is important for water electrolyzers. NiFeO_*x*_H_*y*_ nanoparticles have shown the highest activity towards the OER in alkaline solution.^94^ The size-dependent mass activity of NiFeO_*x*_H_*y*_ nanoparticles towards the OER in alkaline electrolyte is depicted in Fig. 4C. Similar to Pt nanoparticles under ORR conditions, the smaller nanoparticles of 3.9 and 5.4 nm sizes showed higher mass activities compared to larger particles with 6.7 and 8.4 nm in diameter, although the activity does not scale linearly with size. Moreover, the surface-area-specific activities were size independent. From isotope labeling and mass spectrometry experiments the authors concluded that the active centers are located at the near-surface region (∼3 atomic layers) of the nanoparticles and that lattice oxygen atoms are not involved in the OER. In brief, when designing the catalysts for the OER it is important to control the atoms at the near-surface region rather than the bulk atoms.

## Shape effects

Size and composition are two crucial parameters in determining nanoparticle reactivity. Additionally, the structural sensitivity of certain electrocatalytic reactions makes nanoparticle shapes another important aspect to be considered.^95^–^99^ The shape of the particle usually dictates which crystallographic facets are exposed: for instance, octahedra and tetrahedrons expose (111) facets, cubes expose (100) facets, whereas truncated octahedra and cuboctahedra expose a combination of (111) and (100) facets.

**Fig. 5:**
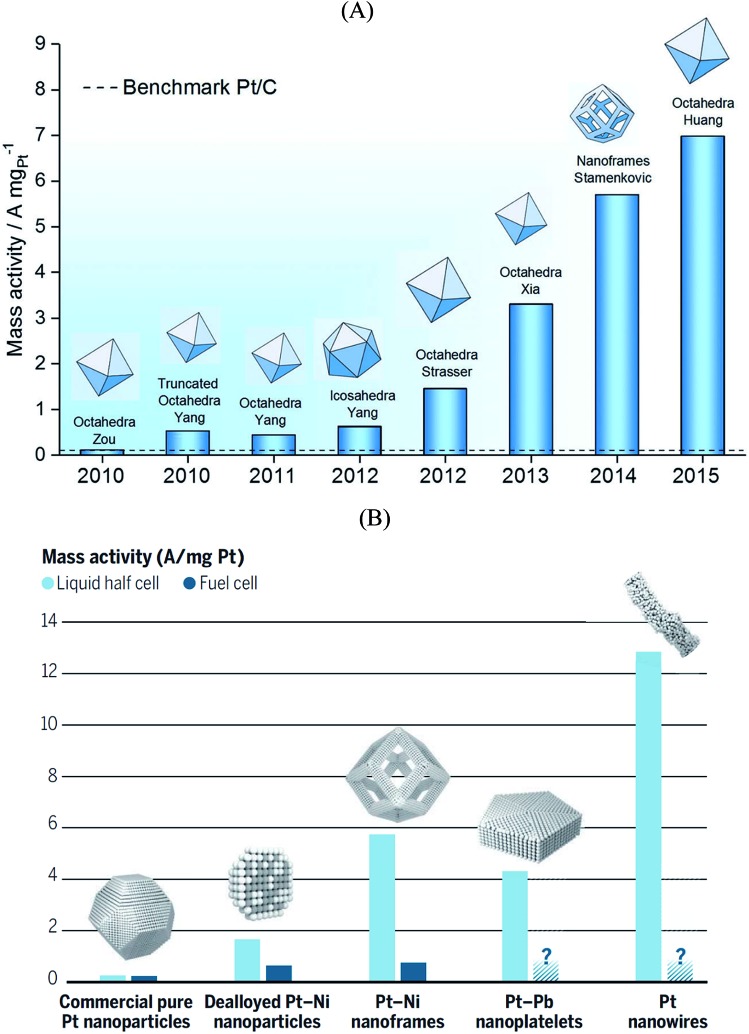
(A) Mass activity of various PtNi nanoparticles measured at 0.9 V *vs.* RHE plotted against their publication year. Reproduced from ref. 11 with permission from Royal Society of Chemistry. (B) Performance of various Pt-based catalysts. Mass activities measured in half-cell setups (light blue) compared with performance in fuel cell (dark blue). Reproduced from ref. 102 with permission from American Association for the Advancement of Science.

**Fig. 6:**
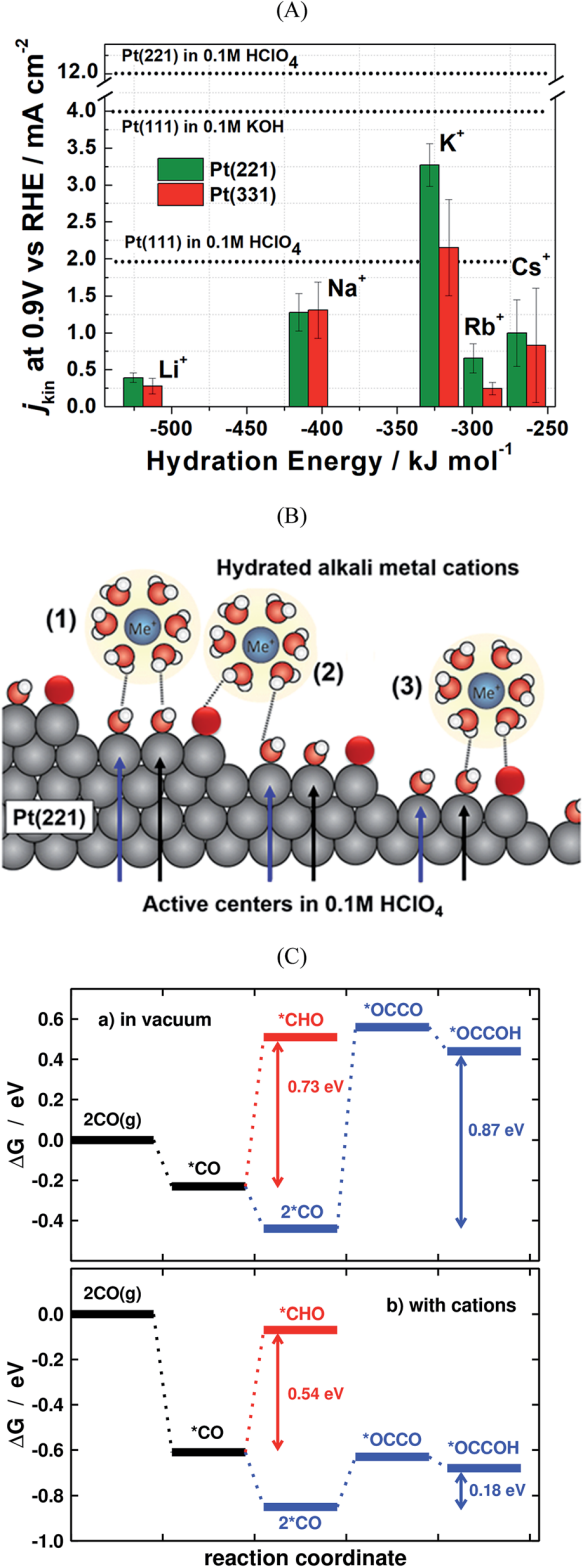
(A) Kinetic current densities of Pt(221) and Pt(331) electrodes in different alkali metal hydroxide solutions as a function of the corresponding hydration energies. Specific activity of Pt(111) in acidic and basic solutions are shown by dotted lines. The activity of Pt(221) in 0.1 M HClO_4_ is also shown with a dotted line for comparison. (B) Schematics of the interaction between water-solvated alkali metal cations and the steps and terraces of Pt(221) surfaces. White, red, and black spheres represent H, O, and Pt, respectively. (A) and (B) are reproduced from ref. 109 with permission from American Chemical Society. (C) Energetics of the first electrochemical steps of CO reduction, both in vacuum and with cations, for the C_1_ and C_2_ pathways on Cu(100) at 0 V *vs.* RHE. Reproduced from ref. 31 with permission from American Chemical Society.

Pt_3_Ni{111} single crystalline surfaces are known to be exceptionally active in catalyzing the ORR reaction.^100^ Over the past decade several studies reported on Pt_*x*_Ni alloyed nanoparticles that resulted in new benchmark mass activities for the ORR. Fig. 5A shows the ORR mass activities of various shapes of Pt_*x*_Ni alloyed nanoparticles. Theoretical studies predict that the octahedral nanoparticles mainly contain {111} facets and show highest mass activity towards the ORR.^101^ As seen in Fig. 5A, the highest mass activity is observed for octahedral Pt_*x*_Ni nanoparticles.^11^ The mass activity of commercial Pt/C catalyst is indicated by a dashed line. Because of the instability of Ni in acidic solutions, Ni atoms are leached out during the ORR activity tests; however, the high ORR activity of the nanoparticles is predominantly maintained. From these observations, one can conclude that after Ni atoms are leached out from the initially {111}-oriented Pt_*x*_Ni surfaces, highly active sites with specific arrangement of Pt atoms are formed that determine the overall catalytic performance. The mass activities reported in Fig. 5A are measured in a half-cell three-electrode rotating disk electrode (RDE) setup. The activity of commercially available Pt/C catalysts from RDE is in good agreement with the activities measured in fuel cells, but the situation is quite different for highly active catalysts. The high performance of Pt_*x*_Ni alloyed nanoparticles has not yet been reproduced in real fuel cells.^102^Fig. 6B shows the mass activities of various nanoparticles measured in liquid half-cell setups and PEM fuel cells. As seen in the graph, translating the high performances observed in liquid half-cell setups into real fuel cells remains a challenge. This is mainly due to the differences in operating environment, structure of the catalyst layers, and operation temperature. Particularly in the case of Pt-based alloyed nanoparticles, the contamination of the membrane due to the de-alloying of the solute metal decreases the performance.^103^,^104^ Conversely, in RDE this does not influence the activity, as the solute metal is dispersed in the electrolyte bulk.

The stability of the particle shape over the lifetime of the catalyst is another important aspect.^98^ For Pt_3_Ni catalysts, a beneficial effect of doping with other transition elements has been reported, both with respect to activity (*cf.*Fig. 5A) and shape stability.^97^ Also, a larger stability of (less active) cuboctahedral Pt_3_Ni as compared to octahedral NPs was reported.^75^

## Solvent and electrolyte effects

The electrolyte composition is another parameter that can substantially modulate the overall catalyst performance.^29^,^105^–^108^ The ORR activities of various single crystalline Pt electrodes in different alkaline solutions have been investigated by Garlyyev *et al.*^109^ Interestingly, the influence of the alkali metal cations is surface structure sensitive. As shown in Fig. 6A, the activity of Pt(111) increases if the electrolyte pH is changed from acidic to basic; however, the trend is reversed for high-index stepped Pt surfaces, such as Pt(221) and Pt(331). This is attributed to the presence of different types of active sites for stepped surfaces compared to pristine (111) surfaces. Fig. 6B illustrates how the alkali metal cations interact with Pt(221) surfaces. As said before, for an optimal ORR activity in acid the sites should have *ca.* 0.1–0.15 eV weaker *OH adsorption energy than Pt(111) terrace sites,^35^ which is found at concave defects or strained (111) terraces.^20^,^62^,^67^,^73^ In alkaline solutions the metal cations somehow modify *OH adsorption so that the optimal binding is found at (111) terraces. Thus, the presence of defects, either concave or convex, results in lower overall ORR performance for Pt electrodes in presence of alkali cations.

To derive the aforementioned *OH adsorption energy criterion for ORR materials screening (*i.e.* Δ*G*XOH – Δ*G*Pt(111)OH = (0.1–0.15) eV), some approximations need to be made. In particular, the identical solvation of the ORR adsorbates on all Pt-based catalysts is assumed, regardless of the lattice constant and alloying elements present in the structure. Solvation has been, therefore, habitually regarded as an adsorbate-dependent but structure- and composition-independent correction. However, recent works showed that this assumption is only suitable for the analysis of overall trends, and should be cautiously used when predicting the ORR activity of new Pt-based materials, as the differences in adsorbate solvation energies can be significant, particularly for *OH, the archetypal ORR intermediate.^110^ The same is true for other materials such as metalloporphyrins.^111^ Here it is worth mentioning that generalized coordination numbers in conjunction with micro-solvation can also be used to calculate average water stabilization values for extended surfaces and nanoparticles.^112^

Additionally, solvation is a crucial factor in studies aimed at breaking scaling relations in solution. As seen in the recent literature,^111^ adsorption-energy scaling relations broken under vacuum conditions can, in some cases, be restored in solution by virtue of solvation. As said before, it is not always advisable to include solvation as an external, constant correction, regardless of the nature of the active site.

Another important factor when modelling electrocatalytic systems are cation and anion effects. Numerous works have shown in the literature that the selectivity of CO_2_ and CO reduction depends on the size and concentration of halides and alkali cations.^113^–^115^ Moreover, in the recent literature it has been shown that cation effects are structure- and potential-dependent.^31^ For instance, DFT calculations show that during CO electroreduction on Cu, the hydrogenation of species containing C–C bonds is substantially enhanced by cations with respect to C_1_ species, justifying the selectivity towards ethylene at low overpotentials. This is illustrated in Fig. 6C, where the energetics of *CO hydrogenation to *CHO and *OCCOH are compared.

Furthermore, the impregnation of catalyst layers with ionic liquids (ILs) can have a beneficial effect on the catalytic activity, especially for those showing high oxygen solubility.^116^ This approach was applied to Pt–Ni nanoframes, resulting in an additional enhancement of their activity.^86^ Oxygen solubility is only one of many aspects, and the modification of the interfacial properties at the triple phase boundary is crucial.^117^ For example, recent systematic studies for Pt catalysts showed that preventing the formation of non-reactive oxygenated species at the catalyst surface is another important factor, and that the blocking of surface sites by ILs must as well be minimized.^118^

## Predicting the selectivity and stability of catalytic centers

While most current research on electrocatalysis focuses on increasing the activity of catalysts, one has to consider two additional factors for the commercial realization of fuel cells and electrolyzers: the stability and selectivity of catalytic centers, as they strongly influence lifetime and efficiency. For instance, investigating the catalytic processes at the H_2_/Air fuel-cell interfaces, Pt nanoparticles supported on nanostructured carbon (Pt/C) are typically used as active material on both the anode and cathode catalyst layers. Two key factors limiting the industrial implementation of this technology can be described as: (i) degradation of Pt/C at elevated temperatures, leading to large performance losses over time, as well as (ii) poisoning of the Pt active sites by *e.g.* CO residues present in the gas phase,^119^ leading to a continuously reduced performance.

Increasing the stability of Pt/C catalysts has been the objective of numerous studies over the past decades. Possible solutions such as improved carbon support stability, improved Pt–C anchoring or thermal treatment have been found through experimental work.^120^ Monitoring the durability of fuel cells is challenging in view of the long operation time. Typical post-mortem analysis by disassembly and analysis of a degraded fuel-cell stack can take months, making it unsuitable for catalyst screening.^41^–^43^,^121^ Accelerated stress tests, performed in an electrochemical half-cell setup (RDE setup), can drastically reduce screening times, but monitoring the impact of different degradation phenomena requires different supporting techniques.^122^

Conversely, a protocol for quick catalyst stability evaluation was proposed by Frydendal *et al.*^123^ By coupling the conventional RDE technique with an electrochemical quartz microbalance and inductively coupled plasma mass spectrometry,^124^ they collected corrosion and mass loss data for RuO_2_ and MnO_*x*_ within a short period of time that provided preliminary guidelines for stable catalyst design. Although only OER catalysts were examined, the principle can be extended to other electrochemical reactions. Importantly, as the method is not capable of long-time stability prediction, further advances are much-needed.

**Fig. 7:**
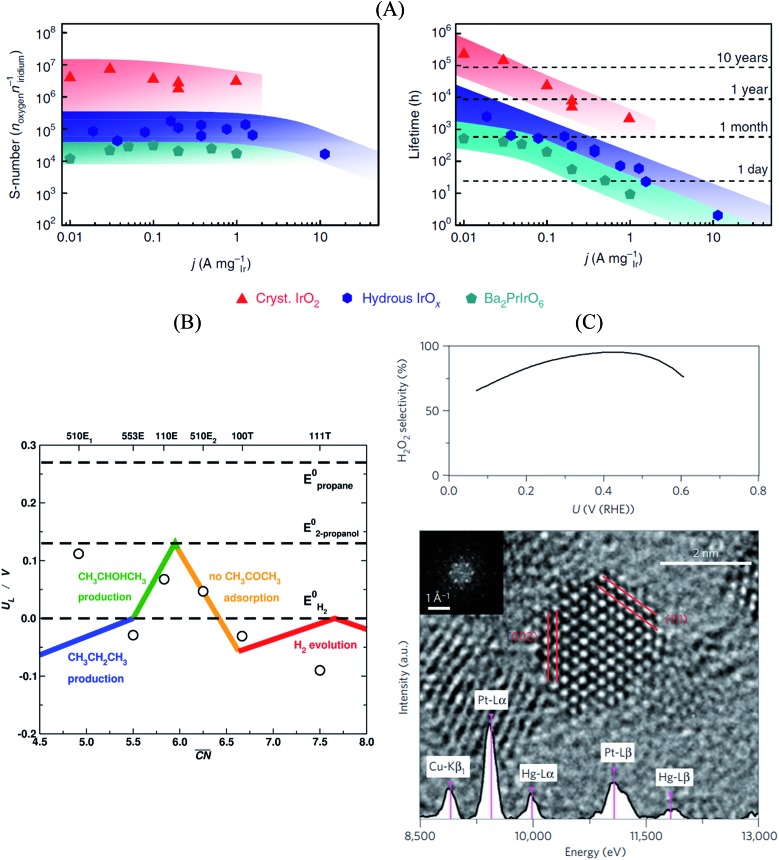
(A) Correlation between S-number (left), lifetime (right) and current density of different Ir-based materials. The stability prediction in a long-time period was calculated using the S-number of the corresponding materials. Figures reproduced from ref. 125 with permission from Nature Publishing Group. (B) Selectivity map of different Pt single-crystal surfaces towards the electrochemical reduction of acetone to isopropanol and propane, as a function of the generalized coordination number of the active centers. Figure reproduced from ref. 70 with permission from Nature Publishing Group. (C) H_2_O_2_ selectivity (top) and TEM image (bottom) of Pt–Hg/C, indicating a selectivity optimum of almost 100% at a potential of ∼0.4 V *vs.* RHE. Figures reproduced from ref. 129 with permission from Nature Publishing Group.

In this sense, a novel stability descriptor proposed by Geiger *et al.*^125^ shows potential improvements over current methods, as it only depends on the dissolution properties of the catalyst material, excluding other typically critical parameters. The so-called stability number (S-number) monitors the ratio of catalytically produced molecules and dissolved catalyst material, giving an indication about the lifetime of the catalyst. The approach was tested and verified for prominent OER catalysts based on Ir, and should be easily extendable to other reactions and materials. The correlation between the S-number, predicted lifetime and the applied current density of different Ir-oxide-based powders is given in Fig. 7A. Remarkably, a long-time prediction of the catalyst stability of up to 10 years was derived from the S-number values, showing its potential for future catalyst design. Moreover, a convenient metric to guide future sustainable OER catalyst design was proposed by Markovic *et al.*^126^ The so-called activity–stability factor (ASF) correlates both the O_2_ generation and the dissolution rate of the catalytically active material.

Regarding the selective oxidation of CO on Pt, Cao *et al.*^127^ recently deposited iron hydroxide clusters on Pt nanoparticles using atomic layer deposition. The system achieves a 100% oxidation of CO over a broad range of temperatures, showing its huge potential as integrated gas purifier during hydrogen fuel-cell operation. Importantly, DFT was used to identify particularly active interfacial structures and captured Pt sites, giving the opportunity to design and tailor catalysts that enable full CO oxidation, thus circumventing CO poisoning during fuel cell operation.

There is another emerging field that requires catalysts with both high activity and selectivity: the electrochemical synthesis of high added-value and commodity chemicals. This area is becoming increasingly important, mainly because it can use excess renewable energy (*e.g.* from wind power) to enable cheap production. On top of that, electrosynthesis can help valorize biomass. The procedure can be applied to various valuable chemicals, as exemplarily highlighted by the work of Xue *et al.*^128^ In their work, they showed that *n*-butylamine, when added into the aqueous electrolyte of a water electrolysis cell, can be selectively oxidized to *n*-butyronitrile. This is remarkable, as typical Ni-, Co- and Fe-based OER catalysts can be used in the cell. In addition, no poisoning was observed at the cathode, where Pt active centers catalyze the hydrogen evolution reaction (HER).

Furthermore, Bondue *et al.*^70^ recently inspected the intriguing electroreduction of acetone on Pt single-crystal electrodes. While the experiments show that Pt(111) and Pt(100) are catalytically inert, Pt(110) and Pt(553) reduce acetone to isopropanol, and Pt(510) does it to propane. Generalized coordination numbers and DFT calculations were used to build the structure-sensitive selectivity map in Fig. 7B. Basically, not only does the activity depend on the geometry of the active sites but also their selectivity. In this case, high coordination sites are not able to adsorb molecular acetone, which renders them inactive, whereas sites with moderate coordination produce isopropanol and those with low coordination are selective towards propane.

Siahrostami *et al.*^129^ used DFT calculations to screen over promising catalysts for the electrochemical H_2_O_2_ production in acidic media. This process is particularly interesting, as it permits an efficient synthesis of H_2_O_2_ from its elements, compared to the conventional energy-intensive, environmentally harmful industrial processes. The catalyst is highly active and selective towards the reaction, while simultaneously being stable during the rough synthesis conditions.^130^ In their work, Siahrostami *et al.* focused on noble metal-based materials, in view of their high stability under reaction conditions. Catalyst screening based on adsorption thermodynamics of the reaction intermediates concluded that PtHg_4_ meets all the requirements for an efficient H_2_O_2_ synthesis, as the surface spatial configuration of Pt and Hg guarantees suitable *OOH binding and avoids *O formation, which implies that O_2_ reduction to H_2_O is hindered. Experimental studies on nanostructured Pt–Hg/C catalysts validated the prediction, with selectivities to H_2_O_2_ close to 100% at certain conditions together with high cycling stability (Fig. 7C).

In summary, considerable progress has been made in the design of more stable and selective electrocatalysts, although trial-and-error attempts often form the basis of these results. Quick prediction of both phenomena, however, significantly accelerates the development of promising catalysts and allows for a favorable compromise of activity, stability and selectivity. As the current computational power increases and new approaches based on *e.g.* machine learning are routinely used,^131^ DFT-based screening will hopefully predict not only more accurate activities but also the selectivity and stability of electrocatalysts. Novel metrics, in line with the experimental “stability number”, would facilitate the prediction of stable catalysts. For instance, there exist descriptors to evaluate the reactivity of lattice oxygen^132^ and activity–stability plots have also been proposed.^51^,^133^ Both approaches may help prevent the undesirable decomposition of oxide catalysts during the OER.

## Concluding remarks

The past two decades witnessed blooming progress in the development of electrocatalysts for clean energy conversion, capitalizing on the advances in experimental and theoretical methodologies^7^,^67^,^134^,^135^ for the study of electrified interfaces. Advancements in the preparation of atomically precise single crystalline surfaces and *in operando* characterization methodologies of surfaces enabled in several cases the identification of catalytically active centers. The emerging field of nanoparticle electrocatalysis with controlled shape and defined surface atom arrangements has benefited from those advancements. Importantly, tuning the size and shape of nanostructured catalysts is shown to provide control over their catalytic activity and selectivity. Taking actual advantage of this enhancement requires that both size and shape are stable over the required lifetime of the catalyst. Another vital parameter to consider in electrocatalysis is the electrolyte composition, as recent studies show its influence on the activity, selectivity, and stability of the catalyst.

All these advances have opened up new, knowledge-based ways to enhance the intrinsic activity of a catalyst instead of using just trial-and-error-based experiments and computational models. Nevertheless, having a highly active and stable electrode is only the first – but very important – step towards commercially viable electrocatalysts: for applications in (electro)chemical syntheses, large turnover rates are required. The same is true for fuel cells: the performance of an MEA at low currents can be to a certain extent predicted by the characterization of catalyst layers in liquid environments using rotating disc electrodes and, at even higher currents, by the floating electrode technique.^136^ For the actual operation in a PEM fuel cell, large areal current and power densities (>1.5 W cm^–2^) are required. Under these conditions, mass transport is crucial. The mass transport behavior depends on the catalyst, its distribution on the support, and the thickness and porosity of the catalyst layer. It has been shown that with respect to mass transport behavior, large nanoparticles (for the same loading) might show considerably poorer performance than 2–3 nm nanoparticles, even if they have excellent activity at low overpotentials.^136^ The role of the mesoscale structure, in particular for the reactant diffusion and re-adsorption of intermediates, was demonstrated in the literature for CO_2_ electroreduction.^137^

In brief, the identification of active surface sites, preparation of stable catalysts with a large number of such sites, and the optimization of the catalyst layer structure are paramount endeavors in contemporary electrocatalyst research. We hope to have shown here that the collaborative interplay of theory and experiments can help in the rational prediction and elaboration of enhanced electrocatalysts.

## Conflicts of interest

There are no conflicts to declare.
